# Inhibition of the RhoA GTPase Activity Increases Sensitivity of Melanoma Cells to UV Radiation Effects

**DOI:** 10.1155/2016/2696952

**Published:** 2015-12-28

**Authors:** Gisele Espinha, Juliana Harumi Osaki, Erico Tosoni Costa, Fabio Luis Forti

**Affiliations:** ^1^Laboratory of Signaling in Biomolecular Systems, Department of Biochemistry, Institute of Chemistry, University of Sao Paulo, 05508-000 Sao Paulo, SP, Brazil; ^2^Centro de Oncologia Molecular, Hospital Sirio Libanes, 01308-060 Sao Paulo, SP, Brazil; ^3^Ludwig Institute for Cancer Research (LICR), 01509-010 Sao Paulo, SP, Brazil

## Abstract

Ultraviolet radiation is the main cause of DNA damage to melanocytes and development of melanoma, one of the most lethal human cancers, which leads to metastasis due to uncontrolled cell proliferation and migration. These phenotypes are mediated by RhoA, a GTPase overexpressed or overactivated in highly aggressive metastatic tumors that plays regulatory roles in cell cycle progression and cytoskeleton remodeling. This work explores whether the effects of UV on DNA damage, motility, proliferation, and survival of human metastatic melanoma cells are mediated by the RhoA pathway. Mutant cells expressing dominant-negative (MeWo-RhoA-N19) or constitutively active RhoA (MeWo-RhoA-V14) were generated and subjected to UV radiation. A slight reduction in migration and invasion was observed in MeWo and MeWo-RhoA-V14 cells but not in MeWo-RhoA-N19 cells, which presented inefficient motility and invasiveness associated with stress fibers fragmentation. Proliferation and survival of RhoA-deficient cells were drastically reduced by UV compared to cells displaying normal or high RhoA activity, suggesting increased sensitivity to UV. Loss of RhoA activity also caused less efficient DNA repair, with elevated levels of DNA lesions such as strand breaks and cyclobutane pyrimidine dimers (CPDs). Thus, RhoA mediates genomic stability and represents a potential target for sensitizing metastatic tumors to genotoxic agents.

## 1. Introduction

Among the broad range of skin cancers, melanoma accounts for less than 2% of skin cancer cases. However, melanoma is the cause of the vast majority of skin cancer-related deaths. According to the American Cancer Society, approximately 76,100 new melanoma cases were diagnosed and approximately 9,710 people were expected to die of this type of skin cancer in the United States in 2014 (http://www.cancer.org/cancer/skincancer-melanoma/detailedguide/melanoma-skin-cancer-key-statistics). The rate of melanoma has been dramatically increasing over the last thirty years, and even more alarmingly the incidence of melanoma is growing in children [1, 2].

Exposure to solar radiation is a major cause of skin cancers [3]. Within the spectrum of electromagnetic radiation comprising the solar spectrum, the ultraviolet (UV) region is considered to be highly genotoxic [4]. UV radiation exposure causes damage to many different biomolecules, but DNA is by far the most affected molecule. The promotion of DNA damage by nonionizing radiation, such as UV light, primarily induces lesions via the direct absorption of photons by DNA bases. The ultraviolet radiation spectrum is divided into UVA radiation (315–400 nm), UVB radiation (270–315 nm), and UVC radiation (100–280 nm). UVB and UVC light induce the formation of cyclobutane pyrimidine dimers (CPDs) and pyrimidine(6-4)pyrimidone photoproducts (6-4 PPs), whereas UVA light primarily causes oxidative DNA damage via the formation of 8-oxo-7,8-dihydroguanine (8-oxoG) and cyclobutane thymidine dimers [5, 6], potentially leading to single-strand breaks and other interstrand cross-links (ICLs) in DNA [7].

UVB radiation, which has been associated with the induction of nonmelanoma skin cancer, is considered to be more carcinogenic than UVA radiation. UVA radiation is more abundant in sunlight and can penetrate deeper into the skin compared to UVB radiation. However, UVA radiation is not significantly absorbed by native DNA and is less efficient in inducing direct DNA damage. UVA radiation might indirectly damage DNA via its absorption by non-DNA endogenous sensitizers and via the formation of reactive oxygen species [8, 9]. UVC radiation, which is generally absorbed by oxygen and ozone in the atmosphere, does not reach the surface of the earth and is less harmful to human's skin. Although UVC radiation does not generate reactive oxygen species, this type of radiation has been found to be highly energetic and has become a useful tool for the destruction of many microorganisms, as it is technically simple to generate high doses of UVC radiation at a wavelength (254 nm) approximating the absorption maximum of DNA [10].

The development of metastatic melanoma from normal melanocytes, which typically adhere to the basal membrane of normal skin, is initiated by the selection of a common acquired benign nevus that exhibits aberrant proliferation and that overcomes cellular senescence, resulting in dysplasia. Subsequently, these cells progress to a superficial spreading stage (radial growth phase, RGP) that is confined to the epidermis, and these cells show low invasive potential. However, RGP cells acquire the ability to invade the dermis (vertical growth phase, VGP) and to metastasize [11, 12]. It has long been suggested that motility is necessary and obligatory for tumor cell metastasis [13]. After passing through the basal lamina, tumor cells migrate through the extracellular matrix over long distances for efficient dissemination via blood and lymphatic vessels. Based on the formation of F-actin-rich protrusions that enable forward extension to adhere to their surroundings followed by contraction of their trailing end, tumor cells use both collective motility and single-cell motility based on in vivo experiments. The formation of membrane protrusions requires actin polymerization, and in invasive tumor cells this signaling pathway is altered to increase motility [14, 15].

Rho-family GTPases have been directly associated with motility and protrusion formation via the activation of signaling targets that direct upstream actin cytoskeleton-modifying proteins. Among the 20 members of this GTPase family, RhoA has been shown to play key roles in cytoskeletal dynamics, such as the regulation of cell adhesion and migration [16]. However, RhoA exerts pleotropic effects on cellular metabolism via the regulation of gene transcription, cell differentiation and proliferation, and the cell cycle, and these effects are particularly obvious during the establishment and development of human and mouse tumors [17].

However, the involvement of RhoA in melanoma cell metastasis following exposure to UV light deserves further exploration and understanding. The first report associating Rho GTPase activity with UV radiation-induced DNA damage in human cells and DNA repair signaling pathways showed that RhoB is an early-response gene induced by DNA damage agents which participates in the initial signaling events in response to genotoxic stress promoted by UVB radiation [18]. Studies have also shown that, in keratinocytes, RhoE acts as a protective factor against UVB radiation-induced damage [19], and it was only recently shown that miR-340 regulates UVB light-induced dendrite formation via the downregulation of RhoA protein and mRNA expression in melanocytes [20]. Moreover, cross talk between DNA damage and cytoskeletal dynamics directly involving RhoA and the regulation of cell proliferation and survival were shown in two reports using bacterial cytolethal distending toxins (CDTs) as cytotoxic agents for the promotion of DNA double-strand breaks, which, in turn, led to ATM- and FEN1-dependent RhoA activation under conditions of carcinogenesis triggered by chronic bacterial infection [21, 22].

Based on this strong experimental evidence, the aims of the present study were to examine the correlations between RhoA activity and DNA damage and repair under genotoxic stress promoted by radiation consisting of each one of the three UV wavelengths (A, B, or C) and to determine whether the modulation of RhoA impacts on the motility, invasiveness, and proliferation of human melanoma cell lines. Thus, the cross talk between RhoA activity and genomic stability may suggest this GTPase as a potential target for the sensitization of melanomas to radio-chemotherapies for cancer treatments [23, 24].

## 2. Materials and Methods

### 2.1. Cell Culture

The human melanoma cell line, which was derived from a metastatic site on a lymph node (MeWo lineage, HTB-65), was obtained from the American Type Culture Collection (Manassas, VA, USA) and was maintained in RPMI-1640 medium (Invitrogen, Carlsbad, CA, USA) containing 10% fetal bovine serum (Cultilab, Campinas, SP, Brazil) at 37°C in 5% CO_2_ in a Sanyo model MCO-19AIC (UV) incubator (Sanyo, Osaka, Japan). MeWo cell clones were generated via transfection (using Lipofectamine) with the packaging cell line ΦNX-Ampho (Phoenix) and a plasmid containing RhoA cDNA (mutated at the V14 position (active RhoA) or at the N19 position (dominant-negative RhoA)) cloned into the retroviral vector pCM (pCLNCX backbone). The plasmids were packaged into retroviral particles contained in the viral vector supernatant (>10^6^ c.f.u.), and these retroviral particles were used to transduce or infect MeWo cells in the presence of 4 *μ*g/mL polybrene (Sigma-Aldrich, St. Louis, MO, USA) for 24 h. Infected cells (clones) were selected in culture medium containing the antibiotic G418 (400 *μ*g/mL) because the pCM vector carries a neomycin resistance gene [25]. Mutant clonal cells were maintained in RPMI-1640 medium (Invitrogen, Carlsbad, CA, USA) containing 10% fetal bovine serum and 100 *μ*g/mL G418.

### 2.2. UV Radiation Treatments

MeWo cells and mutant cells expressing dominant-negative RhoA (MeWo-RhoA-N19) or constitutively active RhoA (MeWo-RhoA-V14) were subjected to UV irradiation using the following procedure: the cell culture medium was replaced with PBS, and the cells were exposed to UV radiation lamps at specific wavelengths corresponding to UVA (365 nm), UVB (302 nm), or UVC (260 nm) radiation for the necessary exposure duration to reach an intensity of 50 KJ/m^2^ UVA, 80 J/m^2^ UVB, or 4 J/m^2^ UVC, respectively. These conditions were determined and monitored using a model VLX-3W dosimeter (Vilber Lourmat, Eberhardzell, Baden-Württemberg, Germany) coupled to specific probes for each wavelength; this instrument displayed an accuracy of +/−5%. Following each treatment, the culture medium was replaced, and the cells were incubated for the indicated periods prior to further analyses.

### 2.3. Generation of Rhotekin-Binding Domain-Glutathione S-Transferase (RBD-GST) Fusion Proteins

*E. coli* (BL21) bacteria were transformed with the plasmid carrying RBD-GST (a kind donation from Gary M. Bokoch of the Scripps Research Institute, La Jolla, CA, USA) via thermal shock. Subsequently, the transformed bacteria were plated on LA medium containing 100 *μ*g/mL ampicillin and incubated at 37°C. A colony of transformed* E. coli* (BL21) bacteria was inoculated into 200 mL of LB medium. The inoculum was incubated for 18 h at 37°C under constant agitation (200 rpm). Subsequently, this culture was inoculated into 2 L of LB medium, and the cell culture was maintained at 37°C under constant agitation until reaching an optical density of approximately 0.6. RBD-GST expression was induced by adding isopropyl *β*-D thiogalactopyranoside (IPTG, 0.5 mM), followed by incubation at 37°C for 2 h, and the cells were recovered via centrifugation (8,000 rpm for 10 min at 4°C). The pellet was resuspended in 20 mL of lysis buffer (50 mM Tris, pH 7.5; 150 mM NaCl; 5 mM MgCl_2_; 1% Triton X-100; 1 mM DTT; 10 *μ*g/mL aprotinin; 10 *μ*g/mL leupeptin; and 1 mM PMSF) and sonicated on ice by applying 8 cycles of 2 min at 50% amplitude and a pulse protocol of 15 sec on and 30 sec off. Following lysis, the suspension was centrifuged at 14,000 rpm for 30 min at 4°C, and the soluble fraction containing the RBD-GST fusion protein was collected. Approximately 12 mL of this soluble fraction was incubated in 500 *μ*L of glutathione-Sepharose 4B resin (GE Healthcare, Pittsburgh, PA, USA) for 90 min at 4°C under constant agitation. Subsequently, the resin containing the bound fusion protein was washed (3,000 rpm for 3 min) 6 times with wash buffer (50 mM Tris, pH 7.5; 0.5% Triton X-100; 150 mM NaCl; 5 mM MgCl_2_; 1 mM DTT; 1 *μ*g/mL aprotinin; 1 *μ*g/mL leupeptin; and 0.1 mM PMSF), and the beads were resuspended in 5 mL of wash buffer containing 10% glycerol, followed by aliquoting and storage at −80°C [26].

### 2.4. RhoA GTPase Activity Assay

To obtain protein lysates, the cells were plated on 10 mm dishes at approximately 60% confluence. Following radiation treatment for the specified durations and at the specified doses, the cells were washed twice with ice-cold PBS and disrupted with RIPA lysis buffer (50 mM Tris, pH 7.2; 1% Triton X-100; 0.5% sodium deoxycholate; 0.1% SDS; 500 mM NaCl; 10 mM MgCl_2_; 1 mM Na_3_Vo_4_; 1 mM NaF; 1 mM PMSF; and 10 *μ*g/mL each of aprotinin and leupeptin) and stored at −20°C. The protein concentration was quantified using the Bradford colorimetric method (Bio-Rad). A 500 *μ*g sample of the total lysate was subsequently incubated in 25 *μ*g of RBD-GST at 4°C for 90 min. Then, the beads were centrifuged at 4°C for 3 min, washed three times with buffer B (Tris buffer containing 1% Triton X-100, 150 mM NaCl, 10 mM MgCl_2_, 1 mM Na_3_Vo_4_, 1 mM NaF, 1 mM PMSF, and 10 *μ*g/mL each of aprotinin and leupeptin), and intercalated via centrifugation at 3,000 rpm for 3 min in a cold room. The active RhoA protein (RhoA-GTP) bound to the glutathione-Sepharose beads was detected via Western blotting [26].

### 2.5. Western Blotting for RhoA

To analyze the obtained proteins, electrophoresis was performed under denaturing conditions using polyacrylamide gels consisting of 5% acrylamide in the stacking gel and 13% acrylamide in the separating gel. The proteins were separated via SDS-PAGE at a constant voltage (120 V) and were then transferred to a nitrocellulose membrane (Merck-Millipore, Billerica, MA, USA) using a dry system (Bio-Rad, Hercules, CA, USA) at 300 mA for 90 min. The membrane was blocked with 5% milk in TBS-T (20 mM Tris, pH 7.6; 137 mM NaCl; and 0.1% Tween) for 1 h with stirring at room temperature, followed by three washes with TBS-T. Subsequently, the membrane was incubated for 3 h at room temperature in a monoclonal primary antibody against RhoA (Santa Cruz Biotechnology, Santa Cruz, CA, USA) diluted in TBS-T. The membrane was incubated in the fluorescent secondary antibody IRDye 680CW for 1 h, and the bands were visualized using an Odyssey Infrared Imaging System (Li-Cor, Bad Homburg, Germany). The obtained bands were quantified using Odyssey V3.0 software (Li-Cor, Bad Homburg, Germany).

### 2.6. Stress Fiber, RhoA, and CPD Staining for Immunofluorescence

On the day before the experiment, the cells were plated on glass coverslips at ~25% confluence, maintained under culture conditions described above and subjected to UV irradiation. The cells were subsequently washed twice with PBS and fixed at room temperature with 3% formaldehyde/2% sucrose/PBS (Phalloidin) or 10% TCA/PBS (RhoA) for 10 min, followed by two additional washes with PBS and permeabilization with PBS containing 0.5% Triton X-100, 6.84% sucrose, and 3 mM MgCl for 5 min on ice. Subsequently, the cells were treated with blocking buffer (PBS containing 3% BSA and 10% FBS) for 30 min at room temperature, followed by incubation for 2 h in an anti-Phalloidin antibody conjugated to Alexa Fluor 488 (Invitrogen, Carlsbad, CA, USA) diluted 1 : 500 in blocking buffer (stress fibers) or in a mouse anti-RhoA antibody (1 : 250; Santa Cruz Biotechnology, Santa Cruz, CA, USA) followed by incubation in an Alexa Fluor 680 anti-mouse antibody (1 : 15,000; Invitrogen, Carlsbad, CA, USA) for 1 h in a dark chamber at room temperature in a humidified atmosphere (RhoA). To stain for CPD, coverslips containing an approximately 80% confluent cell monolayer were UV-irradiated and subsequently collected at 0, 6, 24, or 48 h, followed by fixation with 4% paraformaldehyde, permeabilization with 0.5% Triton X-100, and genomic DNA denaturation in the presence of 2 M HCl. The coverslips were incubated for 2 h in a rabbit anti-CPD primary antibody (Cosmo Bio Co., Ltd., Japan) diluted 1 : 200 in blocking buffer and then for 1 h at room temperature in an Alexa Fluor 568 anti-rabbit secondary antibody. The cells were subsequently mounted on glass slides using VECTASHIELD containing 4′,6-diamidino-2-phenylindole (DAPI; 1 *μ*g/mL). The images were visualized and captured using a Zeiss LSM-510 microscope. Quantitation of the fluorescence per cell was performed using ZEN software (Zeiss, Oberkochen, Germany), and at least 50 cells per condition were individually quantified.

### 2.7. Cell Migration Assay

Approximately 1.5 × 10^6^ cells were plated on 35 mm plates and incubated until reaching 100% confluence. After various radiation exposure treatments, the plates were diametrically scratched using a sterile pipette tip. Cell migration was assessed by comparing the cell invasion area of the scratch at the initial time (0 h) with the cell invasion area of the scratch at the ending time (24 h). Several micrographs were obtained along the scratch at 200x magnification using an inverted Olympus microscope, and the cell migration or invasion measurements were conducted using appropriate tools provided in cell-F software (Olympus, Shinjuku, Tokyo, Japan) [27].

### 2.8. Matrigel Invasion Assay

MeWo cells suspended in serum-free medium were plated in the upper chamber of a BD BioCoat Matrigel Invasion Chamber (BD Biosciences, San Jose, CA, USA) (10^5^ cells in 100 *μ*L) and were allowed to invade for 24 h at 37°C in 5% CO_2_ in the presence or absence of the matrix metalloproteinases (MMP) inhibitor GM6001 (Calbiochem, Billerica, MA, USA) at a concentration of 25 *μ*M. The lower chamber was filled with complete medium as a chemoattractant for cellular invasion. At the end of the experiment, the upper sides of the inserts were scraped with cotton swabs, and the cells on the bottom side of the membrane were fixed in 3.7% formaldehyde, subjected to nuclear staining with DAPI (Sigma), and photographed. Cells were counted at 20x magnification in 10 different optical fields per insert.

### 2.9. Growth Curves

The role of RhoA protein in cell proliferation following UV irradiation was observed using growth curves. MeWo cells and RhoA mutant clones (3.5 × 10^4^) were plated on 35 mm plates at 24 h before treatment. Subsequently, the cells were trypsinized, fixed in a formaldehyde/PBS solution, and counted in a Fuchs-Rosenthal chamber every 24 h for five consecutive days.

### 2.10. Clonogenic Assay

Each cell line used in this study was plated at a low density (2 × 10^3^ cells/plate) on 60 mm plates at 24 h before the radiation treatments. Subsequently, the cells were irradiated as previously described and provided with fresh medium, which was replaced every three days until the cell colonies were visible (approximately 10–12 days). The colonies were fixed with 10% formaldehyde/PBS and stained with a 0.5% crystal violet solution for visualization, followed by manual counting and plate scanning.

### 2.11. Single-Cell Gel Electrophoresis or Comet Assay

Parental MeWo cells and MeWo-RhoA-N19 and MeWo-RhoA-V14 mutant cells were plated at a density of 2 × 10^5^ cells/plate on 35 mm plates 24 h before UVA, UVB, or UVC irradiation. Following each specific treatment, the cells were collected via trypsinization and mixed with 0.5% low-melt agarose at 37°C. This mixture was applied to glass slides covered with a thin layer of 1.5% agarose and incubated at 4°C for 15 min for jellification. The cells were subsequently lysed in lysis solution (10 mM Tris, pH 10; 2.5 M NaCl; 100 mM EDTA; 1% Triton X-100; and 10% DMSO) for 24 h at 4°C. Following lysis, the slides were placed in a horizontal electrophoresis tank, immersed in electrophoresis buffer (300 mM NaOH and 1 mM EDTA), and incubated for 30 min to denature the DNA. The slides were subjected to electrophoresis at 1 V/cm and 300 mA for 30 min. Subsequently, the slides were incubated in neutralization buffer (0.4 M Tris-HCl, pH 7.5) for 15 min and fixed in absolute ethanol for 5 min, followed by DNA staining with 2 *μ*g/mL ethidium bromide and visualization under a fluorescence microscope (Olympus BX51). The results of the DNA damage analysis assay were expressed as the olive tail moment, which was obtained using Komet 6.0 software (Andor Technology, Oxford, UK), and 100 cells per sample were analyzed (50 cells per slide) [28].

### 2.12. Statistical Analysis

The treatments were compared to determine significant differences using Student's *t*-test for paired data, and statistical significance was assumed at *P* < 0.05. ANOVA was used for comparing the means of two or more groups.

## 3. Results

### 3.1. Generation and Characterization of MeWo-RhoA Mutant Clones and Investigation of the Effects of UV Irradiation on Cell Migration and Invasion

In the present study, we used the MeWo cell line, an adherent cell line with fibroblastic morphology derived from the lymph nodes of patients with malignant melanoma [29–32]. This cell line was subjected to retroviral transduction with a construct containing the RhoA-N19 (dominant-negative) or the RhoA-V14 (constitutively active) mutant [33] to obtain clonal lines ectopically expressing each RhoA GTPase variant to interfere with the endogenous activity of RhoA in MeWo cells. Nine MeWo-RhoA-N19 clones and six MeWo-RhoA-V14 clones were isolated, and the migration ability of these cells was tested using scratch wound healing assays in the presence or absence of serum (results not shown). We selected the two most representative clones from each mutation and measured the basal levels of RhoA and RhoA-GTP to examine functionality. We demonstrated that the MeWo-RhoA-N19 mutant cells displayed a reduced basal level of RhoA-GTP compared with the MeWo cells and the MeWo-RhoA-V14 cells, which displayed the highest levels of RhoA activity, as expected (Supplementary Figure S1 in Supplementary Material available online at http://dx.doi.org/10.1155/2016/2696952).

**Figure 1:**
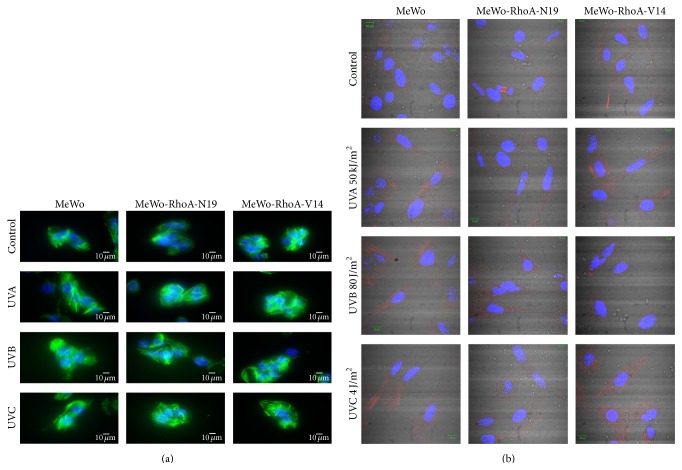
Immunofluorescence analysis was performed on the parental MeWo clone and the MeWo-RhoA-N19 and MeWo-RhoA-V14 mutant clones to evaluate the profile of stress fiber formation and RhoA distribution after damage induced by 50 KJ/m^2^ UVA, 80 J/m^2^ UVB, or 4 J/m^2^ UVC irradiation. A total of 200,000 cells were seeded on 35 mm culture dishes at 24 h before treatment. At 1 h after the given radiation treatment, the cells were fixed in 3% paraformaldehyde/2% sucrose/PBS buffer (a) or 10% TCA/PBS (b) and permeabilized with 0.5% Triton X-100/6.84% sucrose/3 mM MgCl_2_/PBS buffer. Subsequently, the cells were blocked in 3% BSA/PBS for 30 min and incubated in 1 : 500 Alexa Fluor 488 Phalloidin (Invitrogen, Carlsbad, CA, USA) for 1 h at 4°C (a) or in 1 : 250 mouse anti-RhoA antibody (Santa Cruz Biotechnology, Santa Cruz, CA, USA) for 2 h followed by 1 : 15000 Alexa Fluor 680 secondary antibody (Invitrogen, Carlsbad, CA, USA) (b). After washing with PBS, the cells were mounted on coverslips in VECTASHIELD medium containing DAPI, and images were acquired using a Zeiss LSM 510 laser confocal microscope. The photomicrographs are representative of three different fields in two independent experiments.

These MeWo-RhoA clones were exposed to different doses (not shown) of UV (UVA, UVB, or UVC) radiation and examined for stress fiber formation to assess RhoA functionality (Figure 1(a)). The results showed that the RhoA-deficient MeWo-RhoA-N19 clones contained less filamentous actin (F-actin), which was stained with high affinity using Phalloidin, and exhibited a more fragmented morphology than the parental and MeWo-RhoA-V14 clones, particularly after UVC irradiation. The MeWo cell line and the constitutively active RhoA-expressing MeWo-RhoA-V14 clones displayed cytoskeletal features characteristic of physiologic actin function and exhibited normal RhoA levels and stress fiber integrity regardless of the UV treatment applied. Another fundamental morphological characteristic of the MeWo-RhoA mutant cells was the fact that the MeWo-RhoA-N19 cells were thinner and more elongated but the MeWo-RhoA-V14 cells were more spread out and flattened than the parental MeWo cells (Figure 1(b), bright field micrographs). Importantly, the classical and expected cytoplasmic distribution of RhoA was not affected in any of the three cell lines investigated and was not altered by the three different UV treatments applied, as shown in the immunofluorescence microscopy experiments (Figure 1(b), RhoA stained in red).

**Figure 2:**
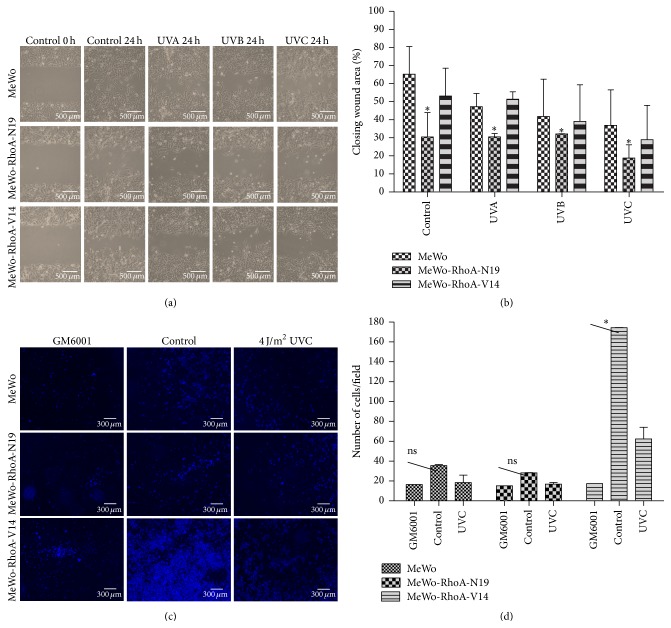
Scratch wound healing and Matrigel invasion assays for MeWo, MeWo-RhoA-N19, and MeWo-RhoA-V14 cells treated with UV radiation. (a) A scratch-like wound was made in a monolayer of cells on 100% confluent plates using a micropipette tip (time zero) prior to irradiation treatment (50 KJ/m^2^ UVA, 80 J/m^2^ UVB, or 4 J/m^2^ UVC). The cells were photographed at 0 and 24 h after treatment at 20x magnification using an inverted microscope (Olympus, Tokyo, Japan), and representative micrographs are shown. (b) Measurements of the initial and final open areas were calculated using cell-F software (Olympus, Tokyo, Japan) and were plotted in bar graphs as the percentage of the closed area. The results are presented as the mean and standard deviation from at least three independent images captured at 24 h after treatment. Two-way ANOVA was performed to compare the RhoA-N19 clone with the two other clones treated according to the same specified conditions. ^*∗*^ *P* = 0.005. (c) Representative micrographs of the Matrigel invasion assay for MeWo, MeWo-RhoA-N19, and MeWo-RhoA-V14 cells untreated (control) or pretreated with 4 J/m^2^ UVC or with 25 *μ*M of a broad-spectrum MMP inhibitor (GM6001). Nuclei of the invasive cells were visualized using DAPI (4x magnification). (d) Quantification of invasive cells shown in 2C. A *t*-test was performed to compare the control cells with the UVC radiation-treated cells from two independent experiments. ^*∗*^ *P* = 0.004; ns, nonsignificant.

Considering the previously described behavior of stress fiber assembly, the effect of UV on the motility of these cells was evaluated using scratch wound healing assays in the presence of 10% serum. The results for the three cell lines highlighted the inhibition (28% maximum) of cell migration following exposure to each of the three UV wavelengths compared with no treatment, and the most pronounced effect was consistently triggered by UVC irradiation (Figures 2(a) and 2(b)). In addition to their expected reduced migration, the RhoA-deficient MeWo-RhoA-N19 cells were less sensitive to the effects of UV irradiation on motility than the cells displaying high levels of RhoA activity. To avoid inaccurate conclusions regarding whether the dominant-negative RhoA-expressing clones were migrating or proliferating within 24 h in the wound healing assays, new migration assays were performed in the presence of two different doses of mitomycin C, and the cells were evaluated after 16 and 24 h. The results showed no differences in cell migration in the presence (Supplementary Figure S2) or absence of mitomycin C (Figure 2) based on a comparison of the three cell lines. This finding confirmed that the MeWo-RhoA-N19 clone was clearly less motile than the other two clones, independent of the presence of the antiproliferative agent mitomycin C.

To determine whether RhoA-dependent sensitivity to UVC radiation treatment also influences the invasiveness of MeWo cells in vitro, the capacity of the MeWo clones to invade through Matrigel was evaluated using Transwell invasion assays (Figures 2(c) and 2(d)). In the absence of UV irradiation, the invasive capacity of the MeWo cells directly correlated with their RhoA activity levels (MeWo-RhoA-V14 > MeWo > MeWo-RhoA-N19). These data were supported by preliminary spheroid invasion assays (multicellular tumor spheroids (MTS) formed via the spontaneous aggregation of 10,000 cells/well and embedded in 3D rat-tail type-1 collagen matrices), showing that the constitutively active RhoA-expressing MeWo clones exhibited an invasive phenotype, in contrast to the RhoA-deficient MeWo clones (Supplementary Figure S3). Notably, pretreatment with a broad-spectrum MMP inhibitor (GM6001) robustly suppressed the invasiveness of the three cell lines, including the highly invasive MeWo-RhoA-V14 cells, indicating that MeWo invasion through Matrigel is MMP-dependent. In agreement with the results of the migration assays (Figure 2(b)), the inhibitory effects of UVC irradiation on cell invasion were clearly the least pronounced in the cells displaying the lowest RhoA activity levels (Figures 2(c) and 2(d)).

### 3.2. The Proliferation and Survival of RhoA-Deficient MeWo-RhoA-N19 Cells Are More Strongly Affected by UV Radiation Than Cells Displaying Normal RhoA Activity

**Figure 3:**
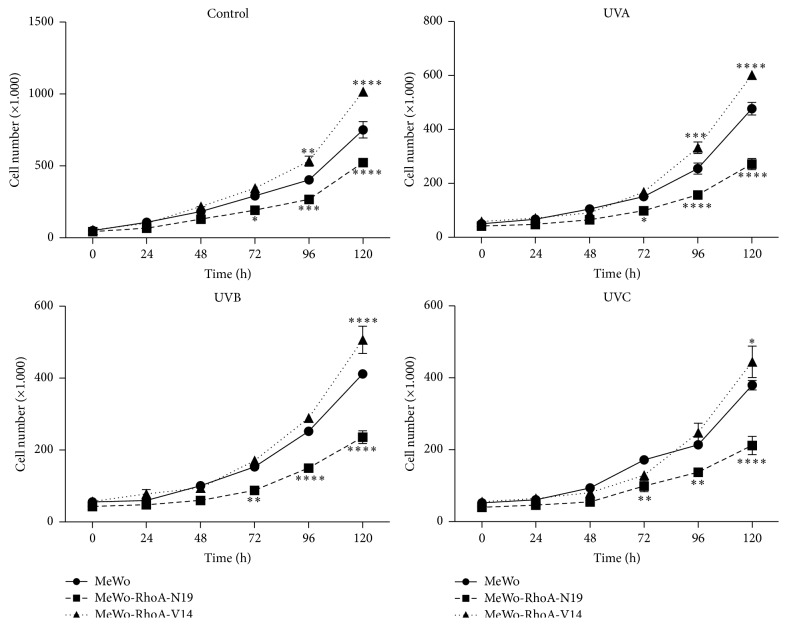
Proliferation curves for the MeWo, MeWo-RhoA-N19, and MeWo-RhoA-V14 clones exposed to genotoxic-equivalent doses of UV radiation. The cells were seeded at a density of 35 × 10^3^ cells per 35 mm culture dish at 24 h before treatment and were exposed to 50 KJ/m^2^ UVA, 80 J/m^2^ UVB, or 4 J/m^2^ UVC radiation. The cells were collected every 24 h for five consecutive days and were counted daily in a Fuchs-Rosenthal chamber. The graphs are representative of three independent experiments, and the standard deviation and statistical significance are shown for only the fifth day. Two-way ANOVA was performed to compare the mutant clones with the MeWo clone treated according to the same specified conditions. ^*∗*^ *P* = 0.01; ^*∗∗*^ *P* = 0.005; ^*∗∗∗*^ *P* = 0.0001; ^*∗∗∗∗*^ *P* < 0.0001.

The measurements of cellular proliferative capacity after any type of genotoxic stress, such as UV radiation of any of the three wavelengths applied, revealed how the cells recovered in response to damage to DNA or other biomolecular structures to escape death or to enter cell cycle arrest. Thus, proliferation curves were generated for the MeWo clone and the two RhoA mutant MeWo clones for five consecutive days following exposure to UVA, UVB, or UVC radiation or no treatment (Figure 3). The initial results showed that the three cell lines responded more effectively as the energy of the applied UV radiation decreased; that is, UVA < UVB < UVC. The MeWo-RhoA-V14 clone exhibited higher proliferative capacity, independent of the treatment, closely followed by the parental MeWo clone; however, these two cell lines, which displayed high levels of RhoA activity, were much more resistant to the deleterious effects of UV irradiation on cell proliferation than the RhoA-deficient clone. These results were confirmed by the cell cycle distribution of the cell population, as analyzed by flow cytometry, which showed a discrete and expected perturbation of the cell cycle distribution in the cells displaying high RhoA activity (Supplementary Figure 4). However, the MeWo-RhoA-N19 cells were the most sensitized after treatment with all three UV wavelengths, and these cells exhibited a reduction in proliferation of approximately 50% compared with untreated cells (Figure 3). This behavior was fully complemented by the flow cytometry results, which showed an accumulation of cells in G1 phase and a concomitant reduction of cells in G2-M phase at only 24 h after UV treatment in the RhoA-deficient clones (Supplementary Figure 4). This delay in the cell cycle suggests that more of these cells are arrested at the G1 checkpoint, likely reflecting inefficient DNA repair.

**Figure 4:**
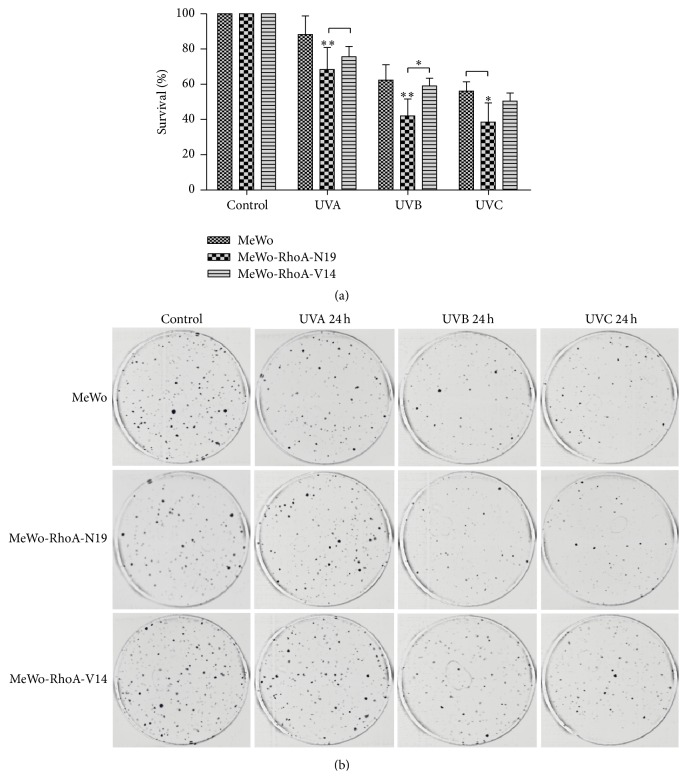
Clonogenic assays showing the highly superior survival of MeWo and MeWo-RhoA-V14 cells, which displayed high levels of RhoA activity, compared with RhoA-deficient MeWo-RhoA-N19 cells after exposure to different types of UV radiation. The bars represent the average and standard deviation of at least three independent experiments. Two-way ANOVA was performed to compare the mutant cells with the MeWo cells treated according to the same specified conditions. ^*∗*^ *P* < 0.01; ^*∗∗*^ *P* < 0.005.

When examined for a longer period (15 days) and when seeded at a much lower density (2,000 compared to 50,000 cells) in colony formation assays, the proliferation and survival capacities showed similar results (Figure 4). Thus, cells exhibiting high levels of RhoA activity (MeWo and MeWo-RhoA-V14) are more resistant to UVA, UVB, or UVC irradiation, resulting in enhanced survival, whereas RhoA-deficient MeWo cells (MeWo-RhoA-N19) are more sensitive to UV irradiation, resulting in reduced survival. The different chemical and physical effects of the three UV wavelengths applied apparently equivalently affected the three cell lines, as each respective cell line responded similarly to all UV radiation treatments.

Consistent with cell proliferation, acute cell death, particularly apoptosis, was evaluated and Annexin V labeling experiments were performed on the same cell lines under the same treatment conditions [34]. As a positive control, H_2_O_2_ was used to produce approximately 50% apoptotic cells. UVA, UVB, or UVC radiation treatment resulted in higher apoptosis in MeWo-RhoA-N19 cells than in MeWo and MeWo-RhoA-V14 cells, which exhibited even lower apoptosis than the parental MeWo cells (Supplementary Figure 5). As the loss of plasma membrane asymmetry is an early reversible event in apoptosis that results in the exposure of phosphatidylserine (PS) residues on the outer plasma membrane [35], these preliminary results show that RhoA deficiency increases the sensitivity of MeWo cells to UV irradiation and renders these cells more susceptible to apoptotic cell death.

### 3.3. MeWo Cells Displaying High RhoA Activity Are Much Less Affected by UV Radiation-Induced Damage, Such DNA Strand Breaks and CPDs, and Exhibit More Efficient DNA Repair Than RhoA-Deficient MeWo Cells

**Figure 5:**
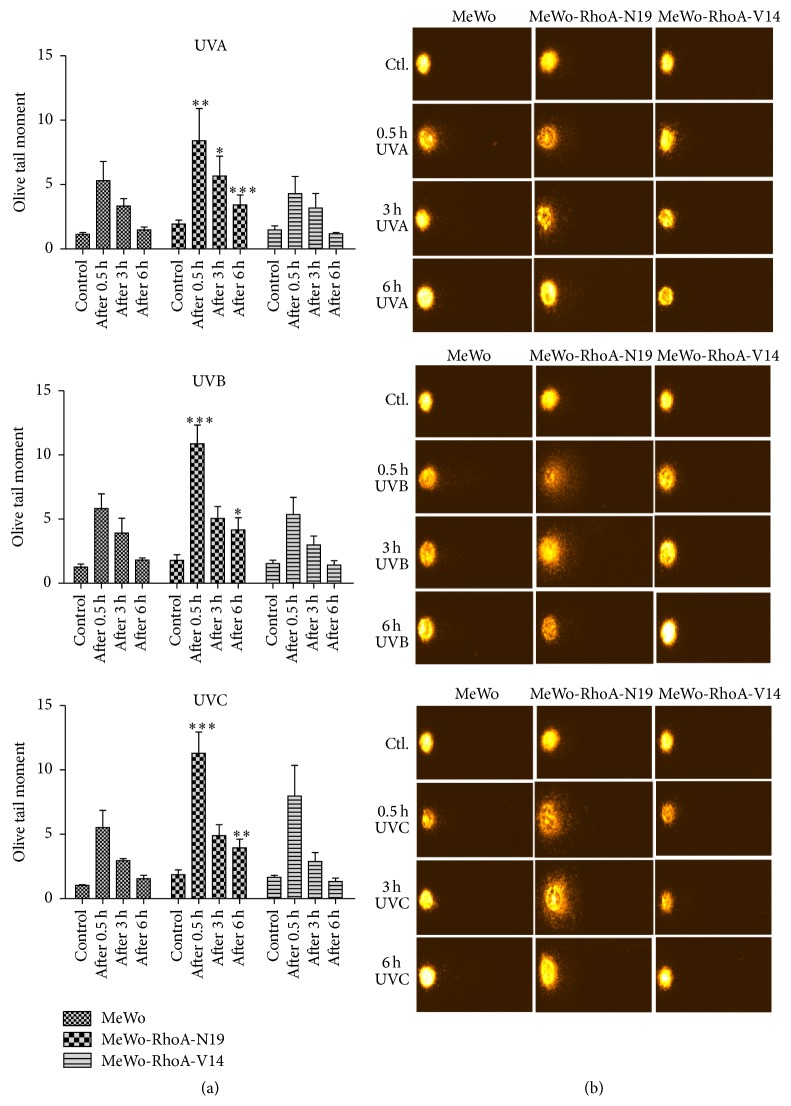
Alkaline comet assays showing DNA single- and double-strand break formation and DNA repair kinetics up to 6 h after exposing MeWo, MeWo-RhoA-V14, and MeWo-RhoA-N19 clonal cells to UVA, UVB, or UVC radiation. In these experiments, control (Ctl.) refers to 0 h or non-UV radiation treatment. A total of 2 × 10^4^ cells were plated on 35 mm culture dishes 24 h before the treatment, followed by trypsinization, suspension in low-melting point agarose, and spreading onto glass slides. After 2 h of electrophoresis in an appropriate buffer, the cell nuclei were stained with ethidium bromide (see details in Materials and Methods), as shown in (b), and many different parameters were acquired using a Nikon microscope controlled by Komet 6.0 software (Andor Technology, Oxford, UK). The most relevant parameter, the olive tail moment, was used to quantify DNA damage and repair in the cells under these conditions (bar graphs in (a)). The graphs represent the average and standard deviation of at least three independent experiments, and the statistical significance of the results was obtained by comparing the effects of different treatments between the MeWo cells and the mutant cells using two-way ANOVA. ^*∗*^ *P* < 0.01; ^*∗∗*^ *P* < 0.001; ^*∗∗∗*^ *P* < 0.0001.

Previous results showed an evident association between reduced proliferative ability and cellular recovery from UV radiation-induced damage, as reflected by the reduced levels of RhoA activity in the MeWo-RhoA-N19 mutant clones. The multiple DNA lesions promoted by UV radiation are well known; therefore, we next explored another potential correlation between DNA damage and RhoA activity in MeWo melanoma cells. For this investigation, we used the alkaline comet assay, which is a general assay for DNA damage, to detect both single and double DNA strand breaks after exposure to the three UV wavelengths (Figure 5). Moreover, using kinetics experiments to detect damage over time, it is possible to infer repair ability over a period of up to 6 h after the irradiation of the cells. For example, the improved ability of MeWo-RhoA-V14 clones to repair UV radiation-promoted DNA damage has been observed, as these cells completely recover to the initial conditions by 6 h after treatment. Additionally, in MeWo cells displaying high levels of RhoA activity, this recovery capacity is highly similar. However, the RhoA-deficient MeWo-RhoA-N19 clone exhibited higher basal damage under the control conditions (minimum of 30% more damage), peaking at 30 min after UV radiation and increasing to approximately 40–50% of that in the other two clones. Intriguingly, the RhoA deficiency of these cells likely reflects their inability to recover from DNA damage up to 6 h after treatment or even to prevent the accumulation of these lesions over time (Figure 5).

**Figure 6:**
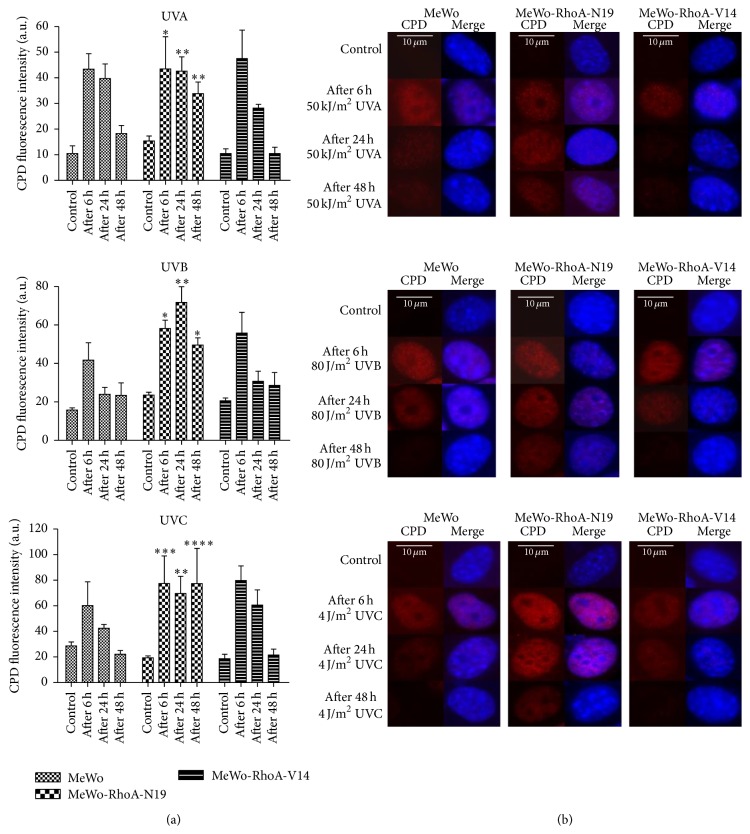
Immunofluorescence analysis for the detection of cyclobutane pyrimidine dimers (CPDs) generated in the MeWo tumor cell line and in the MeWo-RhoA-N19 and MeWo-RhoA-V14 clones after treatment with UV radiation. Coverslips containing an approximately 80% confluent cell monolayer were exposed to UV radiation and collected after 0 (control or non-UV radiation treatment), 6, 24, or 48 h, followed by fixation with 4% paraformaldehyde, permeabilization with 0.5% Triton X-100, and genomic DNA denaturation in the presence of 2 M HCl. The coverslips were incubated for 2 h in an anti-CPD primary antibody (diluted 1 : 200) and then for 1 h at room temperature in an Alexa Fluor 568 secondary antibody (a). Approximately 50 single cells were photographed at 100x magnification and were quantified in sequence using Zen software (Zeiss). The bars represent the average and standard deviation from three independent experiments (b). Two-way ANOVA was performed to compare the mutant clones with the MeWo clone treated according to the same specified conditions. ^*∗*^ *P* = 0.01; ^*∗∗*^ *P* = 0.005; ^*∗∗∗*^ *P* = 0.0001; ^*∗∗∗∗*^ *P* < 0.0001.

The DNA lesions promoted by different UV wavelengths and detected using alkaline comet assays indicate a strong correlation between RhoA activity and DNA damage and repair. Thus, we further measured the formation of CPDs, which are specific, highly toxic, and mutagenic DNA lesions promoted by UV radiation. Using a specific antibody to detect CPDs in the nucleus of the three cell lines, which was delimited based on nuclear staining using DAPI via confocal microscopy, we quantified the DNA lesions after UV irradiation for different periods (Figure 6(a)). Consistent with the results of the comet assay, we observed that the RhoA-deficient MeWo-RhoA-N19 cells exhibited higher levels of CPDs, particularly after UVB or UVC irradiation, and the most striking results were the accumulation of CPDs up to 48 h after either UVA, UVB, or UVC irradiation. Alternatively, the MeWo and MeWo-RhoA-V14 cells exhibited lower CPD staining and greater recovery at 24 h after all three treatments. All of these results were clearly observed directly on the micrographs shown in Figure 6(b), showing a direct correlation between RhoA activity and the function of the nucleotide excision repair (NER) pathway, which is the main pathway responsible for the recovery from CPD lesions.

## 4. Discussion

Approximately ten years ago, the biochemical functions of RhoA (and the typical GTPases) were associated with the regulation of the actin cytoskeleton, the microtubule cytoskeleton, gene expression, and certain uncommon enzymatic activities (involving lipid metabolism and ROS generation). These GTPases were responsible for biological functions such as cell cycle control (G1 progression, mitosis, and cytokinesis), cell morphogenesis (cell-cell interactions and cell polarity), and cell migration (movement and directional sensing) [36]. However, recently, novel RhoA functions similar to those of the Ras homolog were found to be regulated by reactive oxygen species [37, 38], and this regulation may be particularly relevant to some pathological conditions, such as genotoxic stress-induced DNA damage [39]. Thus, RhoA and certain subfamily members were reported to mediate genomic stability or integrity via their indirect involvement in DNA repair mechanisms [18, 19, 21, 22]. Additionally, it was recently shown that RhoA activation is mediated by its physical interaction with the OGG1 protein, a key enzyme in the DNA repair of 8-oxoG modifications [40].

Thus, taking advantage of the well-known roles of RhoA (and other GTPases) in the regulation of actin polymerization and in the metastasis of many types of aggressive tumors [13, 15, 16], including melanomas [11], and considering that the mutagenic effects of UV radiation on melanocytes and keratinocytes trigger metastasis [12, 41], we explored the potential cross talk between the small GTPase RhoA, UV damage and melanoma cell migration, invasion, proliferation, and DNA repair.

These studies were performed in the human metastatic melanoma cell line MeWo upon exposure to three different wavelengths of UV light, as the oxidative stress generated via UVA and UVB radiation and the high energy of UVC radiation induce direct electron transfer/rearrangements in DNA, resulting in serious consequences for the cell cycle [42, 43]. First, we generated cellular models of MeWo cells expressing either constitutively active RhoA (MeWo-RhoA-V14) or deficient RhoA (MeWo-RhoA-N19) for comparisons with parental MeWo cells. These clones were characterized using three different methodologies: (i) pull-down assays, which showed higher levels of RhoA-GTP and stronger responses to serum deprivation for the MeWo and MeWo-RhoA-V14 clones than for the RhoA-deficient MeWo clones (Supplementary Figure 1); (ii) Phalloidin labeling of F-actin fibers, which showed reduced levels of stress fibers and shortened and fragmented cell morphology in the MeWo-RhoA-N19 clones compared with the other cell lines (Figure 1(a)), despite normal subcellular RhoA distribution (Figure 1(b)); and (iii) monolayer migration and 3D matrix penetration assays, which showed reduced motility and invasion capacity of MeWo-RhoA-N19 clones compared with the parental and MeWo-RhoA-V14 clones under control conditions in the presence of serum (Figure 2 and Supplementary Figure S3).

Second, the effects of UV radiation treatment on the three cell lines with respect to stress fiber formation, cell shape, RhoA distribution, migration, and invasion were examined to confirm the deleterious effects of these genotoxic stressors. We observed that UVA, UVB, or even UVC irradiation did not lead to any detectable change in the cell edge shape or the stress fiber morphology in the MeWo or MeWo-RhoA-V14 cells (Figure 1). The migration of these cells was slightly reduced after 24 h, particularly under UVB or UVC radiation treatment (Figure 2(a)), and their invasion capacity was slightly reduced by UVC radiation treatment (Figure 2(b)); these effects were not strictly dependent on the actin-myosin cytoskeleton and were potentially caused by many other factors [44]. Conversely, negative effects on stress fiber morphology and content were observed in RhoA-deficient MeWo-RhoA-N19 clones (Figure 1(a)), very likely reflecting their reduced motility (Figures 2(a) and 2(b)) and invasion ability (Figures 2(c) and 2(d)). The three evaluated cytoskeletal features (Phalloidin staining of actin-myosin fibers, cell edge shape, and RhoA subcellular distribution) are in agreement with each other and with the biological responses of motility and invasiveness characteristic of the aggressive phenotype of melanoma cells and, moreover, with the modulation of RhoA activity. Corroborating the effects of UV radiation treatment on cell migration, a significant reduction in the MMP-dependent invasive capacity of the MeWo cells was observed in all experimental groups following UVC radiation treatment. However, this suppressive effect appeared to be less pronounced in the RhoA-deficient MeWo-RhoA-N19 clones, and this result supports the hypothesis that the inhibitory effects of UV radiation on melanoma cell invasion are partially dependent on RhoA activity.

We next confirmed the well-known antiproliferative effects of UV radiation on melanomas and the potential role of RhoA modulation in this process [17, 24]. Growth curves and colony formation assays confirmed the higher resistance of MeWo-RhoA-V14 and MeWo cells (to a lesser extent) to UV radiation treatment compared with RhoA-deficient cells (Figures 3 and 4), as the MeWo-RhoA-V14 and MeWo cells recovered even after UVB and UVC radiation-induced damage. The opposite effects were observed for the RhoA-deficient clones; that is, these cells exhibited approximately 50% higher sensitivity to UV radiation-induced damage.

As previously reported, these results likely reflect that RhoA affects the efficiency of DNA repair mechanisms [18, 40]. Thus, to assess DNA integrity and specific UV radiation-promoted damage, we performed alkaline comet assays and CPD formation experiments. We showed that the inability to remove damage over time clearly reflects cell proliferation via the modulation of RhoA activity-proficient and activity-deficient MeWo clones. Measurements of single- and double-strand breaks showed that cells displaying high RhoA activity exhibit less damage at 0.5 h (peak) after treatment with the three types of UV radiation and exhibit more efficient repair, completely recovering to the basal levels after 6 h. In contrast, dominant-negative RhoA-expressing cells showed an accumulation of damage from 0.5 to 6 h after injury induced by UVA, UVB, or UVC radiation (Figure 5). Intriguingly, the levels of CPDs peaked at approximately 6 h after UV radiation in all three cell lines, irrespective of RhoA activity, but these lesions were almost completely removed after 48 h in cells exhibiting high levels of RhoA activity. In addition, in the RhoA-deficient MeWo cells (MeWo-RhoA-N19 clones), the accumulation of CPD lesions remained high for up to 48 h, independently of treatment with UVA, UVB, or UVC radiation. Thus, this specific UV radiation-induced damage accumulates in the cellular background of low RhoA activity (Figure 6).

As predicted from the results of other previous studies and confirmed in the present study, the higher the RhoA activity the more efficient the DNA repair; this phenomenon is common to many human tumor cells [45]. UV radiation induces DNA damage, such as single-strand breaks, pyrimidine dimers, and 6-4 PPs, which induce mutations that are characteristic of the promotion, establishment, and development of tumors [46]. These forms of damage are typically repaired by the NER cascade [47], and failures in this repair machinery result in many diseases, such as xeroderma pigmentosum [3]. Thus, in the present study, we established a strong correlation between RhoA activity and the efficiency of the repair of UV radiation-induced damage to melanoma cells, suggesting that NER pathway function might be affected by RhoA-transduced signals that activate cellular responses, such as gene transcription, cell proliferation, and cell death [17]. Although additional molecular studies are needed, RhoA represents a potential target for UV radiation-induced carcinogenesis in skin [48], as well as for gamma radiation-induced damage in cervix carcinomas, where RhoA was also shown to mediate double-strand breaks repair [49]. Similar results, in the same cellular models, were also found for the Rac1 GTPase [50] again suggesting actin cytoskeleton remodeling signals towards the nuclear machineries in charge of the genomic stability.

## Supplementary Material

Supplementary materials include five figures containing images, micrographs and graphs representing complementary experimental approaches, which results were cited and discussed in this text.
