# Supplementary material for: Inhibition of the RhoA GTPase Activity Increases Sensitivity of Melanoma Cells to UV Radiation Effects
Source: Oxid Med Cell Longev. 2015 Dec 28;2016:2696952. doi: 10.1155/2016/2696952 (PMC4707346; doi:10.1155/2016/2696952)
Supplement: Supplementary file 1 — Supplementary materials include five figures containing images, micrographs and graphs representing complementary experimental approaches, which results were cited and discussed in this text. [file 2696952.f1.pdf]

SUPPLEMENTARY MATERIAL

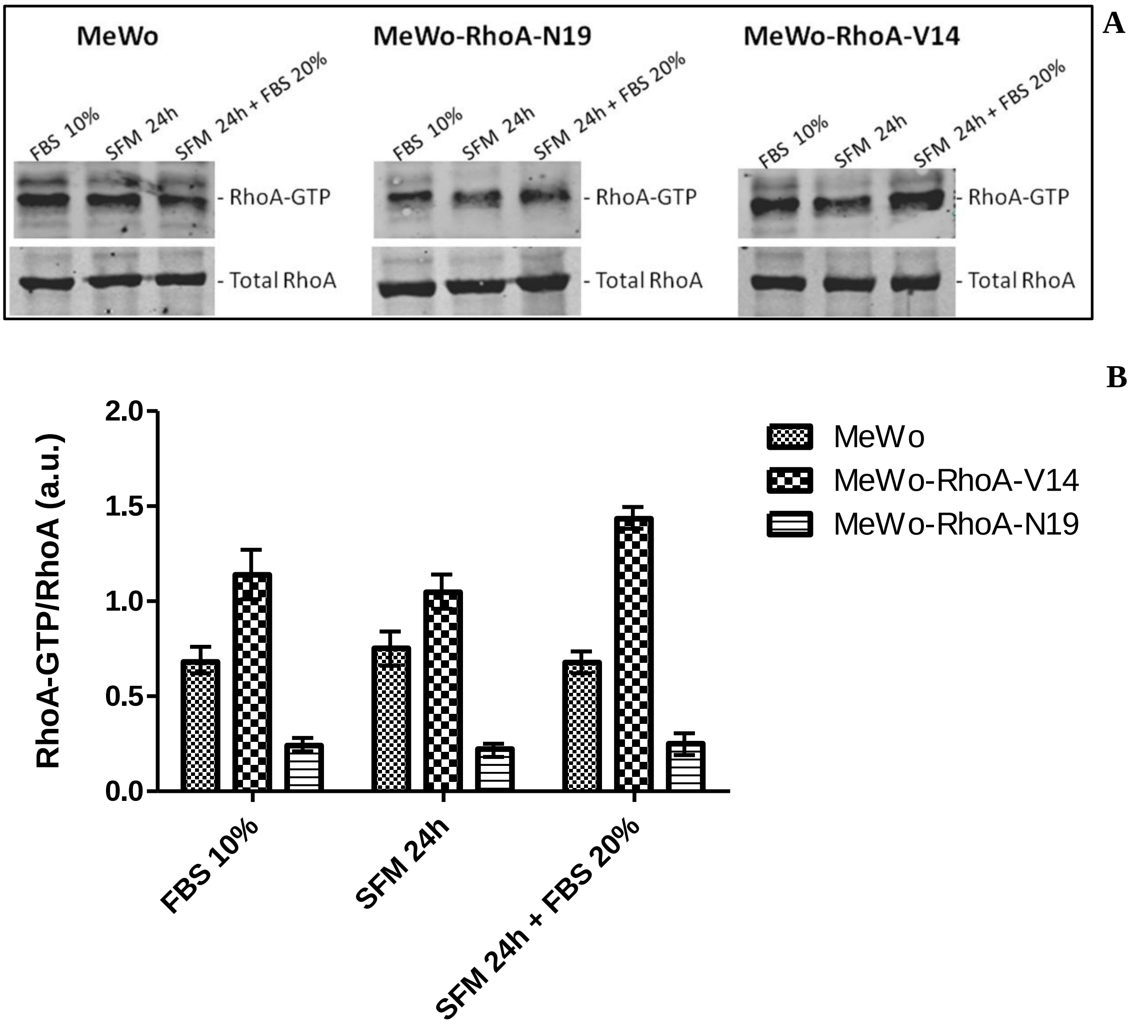

Supplementary Figure S1. Pull-down assays for the measurement of the basal levels of RhoA-GTP in the parental and mutant MeWo clones. A) The three cell lines cultured under exponential growth conditions in 10% fetal bovine serum (FBS) were starved in serum-free medium (SFM) or maintained in serum-containing medium for 24 h and subsequently stimulated or not with 20% FBS to assess RhoA activity. All immunoblots were exposed to a monospecific anti-RhoA antibody (Santa Cruz Biotechnology, Santa Cruz, CA, USA), and the results are representative of two similar

independent experiments. Additionally, the Western blots of the parental, MeWo-RhoA-V14 and MeWo-RhoA-N19 clones were systematically examined using the same gel and were exposed together using the Odyssey Infra-Red Scanner; therefore, there were no differences in the exposure time between the samples. B) Total and active (GTP bound) RhoA bands were quantified by the Odyssey Infra-Red Scanner Software V3.0 and shown as the RhoA-GTP/Total RhoA rate in bars graph as average +/- standard deviation.

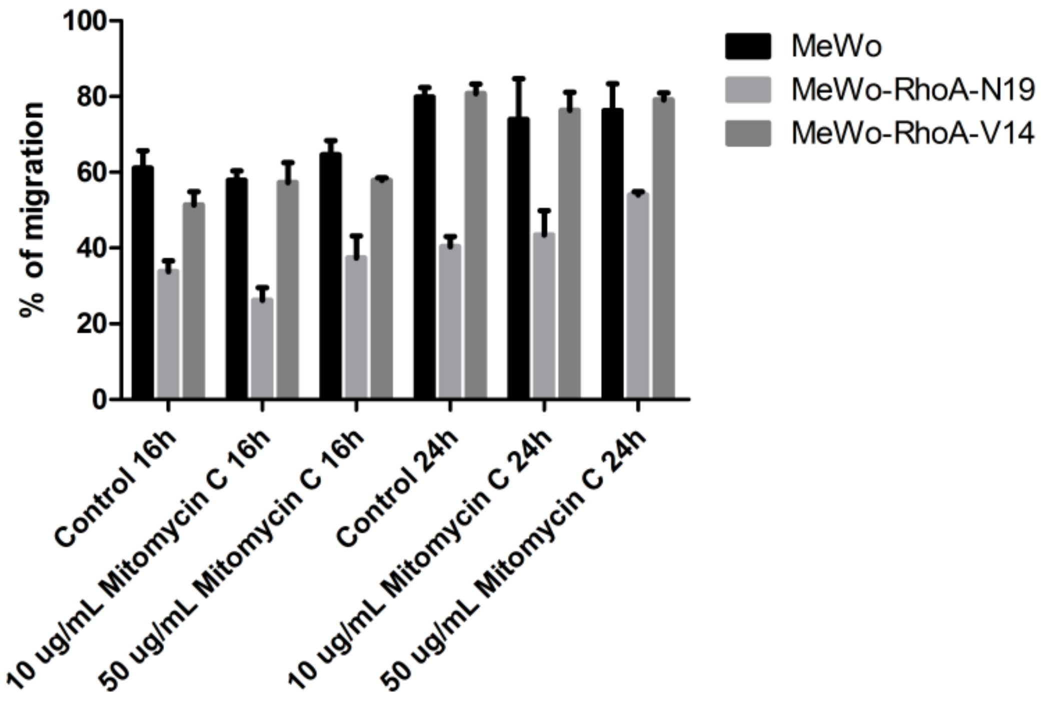

Supplementary Figure S2. Scratch wound healing assay for MeWo, MeWo-RhoA-N19 and MeWo-RhoA-V14 cells treated with Mitomycin C at different doses and durations. A scratch-like wound was made in a monolayer of cells on 100% confluent plates using a micropipette tip (time zero) prior to treatment with 10 or 50 μg/ml Mitomycin C. The cells were photographed at 0, 16 and 24 h after treatment at 20x magnification using an inverted microscope (Olympus, Tokyo, Japan). Measurements of the initial and final open areas were calculated using cell-F software (Olympus, Tokyo, Japan) and were plotted in bar graphs as the percentage of the closed area relative to the control or time zero condition. The results are presented as the average and standard deviation of five images captured after 16 or 24 h from two independent experiments.

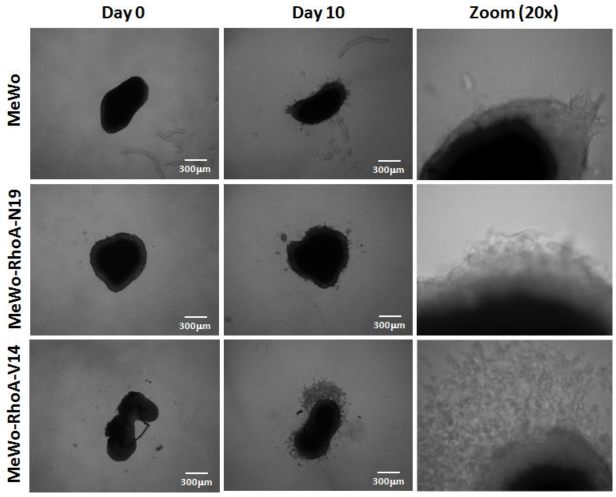

A

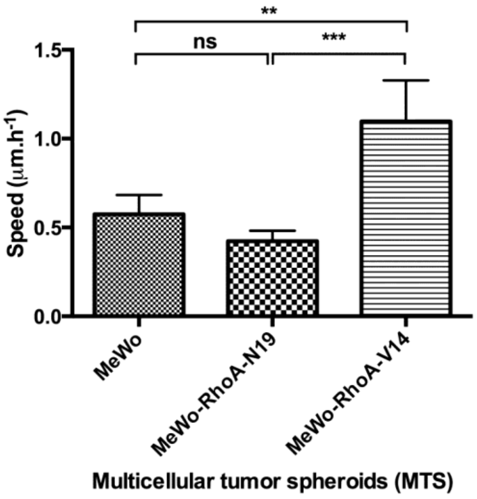

B

Supplementary Figure S3. Spheroid invasion assay showing that the MeWo clones expressing constitutively active RhoA exhibit an invasive phenotype. A) Representative images of multicellular tumor spheroids (MTS) formed via the spontaneous aggregation of 10,000 MeWo, MeWo-RhoA-N19 or MeWo-RohA-V14 cells/well embedded in 3D rat-tail type-1 collagen matrices (1.5 mg/ml, BD Biosciences) for up to 10 days. Briefly, MeWo cells were suspended in complete medium and plated in 24-well plates (Corning) precoated with a 5 mm layer of 1% agarose. After 5 days, a single MTS spontaneously aggregated in each well and was embedded in a collagen matrix in new 24-well plates. B) The average invasion rate was obtained by dividing the distances from the MTS border travelled by 25 leader cells/spheroid by the incubation time. n= 4 MTSs per group. **P<0.01; ***p<0.001 based on two-way ANOVA; ns = non-significant. The error bars indicate the s.e.m.

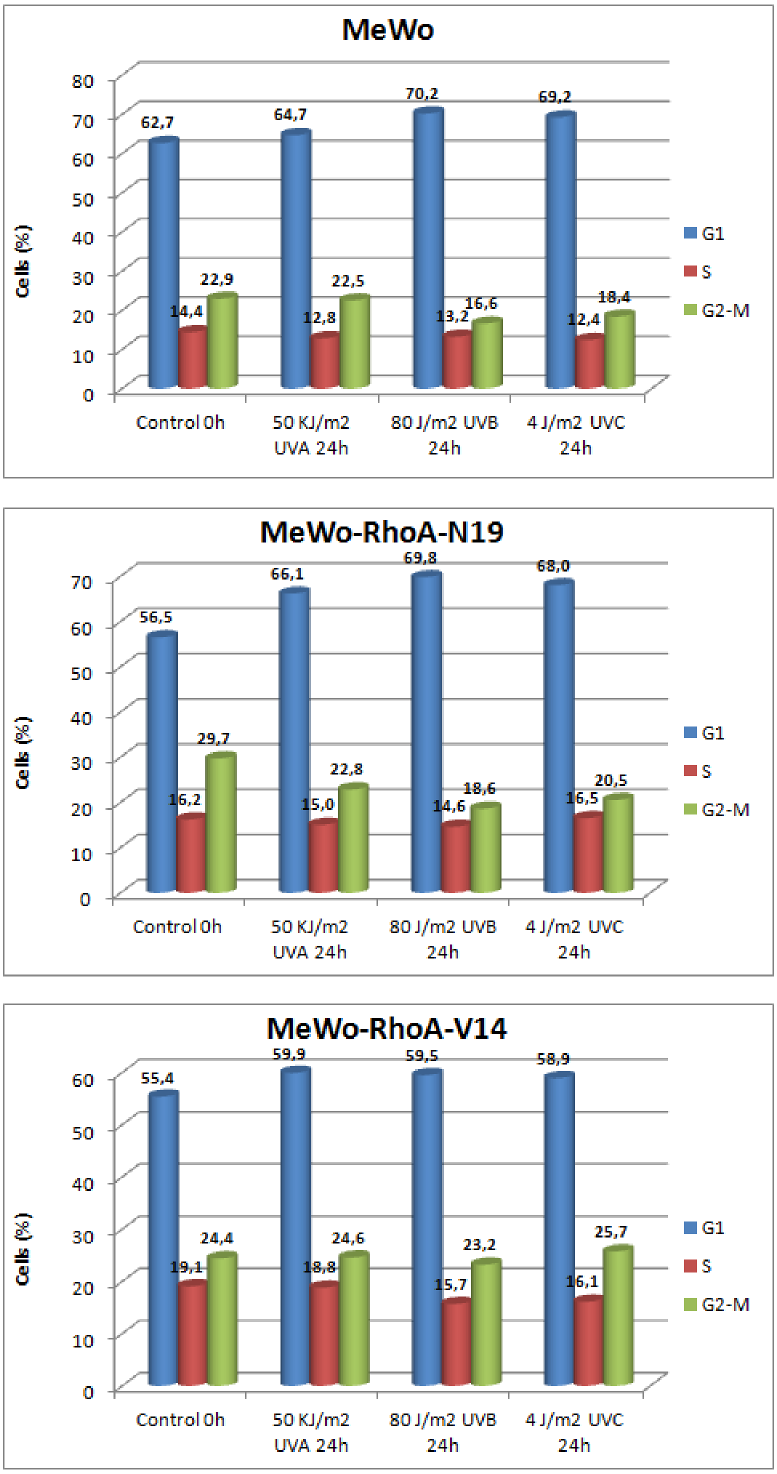

Supplementary Figure S4. Cell cycle distribution of asynchronous MeWo cells and of MeWo-RhoA-V14 and MeWo-RhoA-N19 cells after exposure to the three types of UV radiation. A total of 50×10^3 cells were plated on 35-mm dishes at 24 h before

treatment in PBS with one of the three types of UV radiation according to the doses shown in the figures. The cells were maintained in 5% CO_2 at 37°C, and after 24 h, the cells were trypsinized and resuspended in buffer solution containing propidium iodide (20 µg/mL) for 30 min. Data acquisition was performed using a Guava cytometer (Merck- Millipore, Billerica, MA, USA) considering $10x10^3$ events, and analysis was performed using WinMDI 2.9 software. The results are presented as the average of three independent experiments after discarding the sub-G1 and hyperploid cell populations (typically less than 10% of all cells in each replicate).

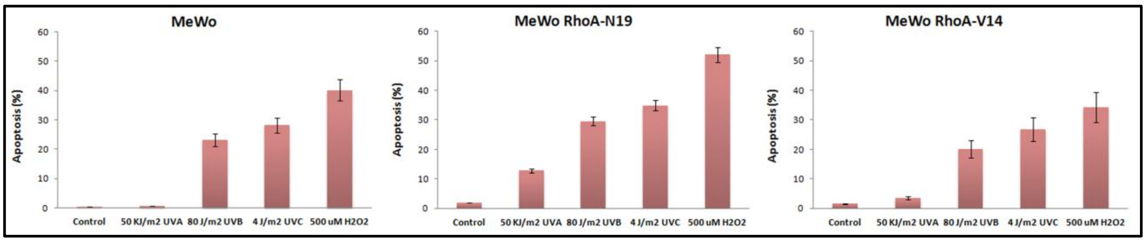

Supplementary Figure S5. Analysis of apoptosis based on Annexin-V labeling of MeWo cells as well as dominant-negative (RhoA-N19) and constitutively-activate (RhoA-V14) RhoA-expressing cells. The cells were plated at 24 h before treatment on 35-mm plates at a density of $50x10^3$ cells. The cells in PBS were treated with UV radiation and were maintained in 5% CO_2 at 37°C. After 120 h, the cells were trypsinized and resuspended in Annexin binding solution, followed by incubation for 30 min in propidium iodide (20 µg/mL) and for 15 min in Annexin-V (sc-4252, Santa Cruz Biotechnology, Santa Cruz, CA, USA). Data were acquired using a Guava cytometer (Merck- Millipore, Billerica, MA, USA) ($10x10^3$ events), and analysis was performed using WinMDI 2.9 software. The results represent the average of two independent experiments.
